# Impact of urban biodiversity and climate change on children’s health and well being

**DOI:** 10.1038/s41390-024-03769-1

**Published:** 2024-12-21

**Authors:** Hana Seastedt, Jackson Schuetz, Alexandra Perkins, Morgan Gamble, Aki Sinkkonen

**Affiliations:** 1 Sean N. Parker Center for Allergy and Asthma Research, Stanford University, Palo Alto, CA USA; 2 Loyola Stritch School of Medicine, Maywood, IL USA; 3 Department of Pediatrics, University of Washington, Seattle, WA USA; 4 Natural Resources Institute Finland (Luke), Turku, Finland

## Abstract

**Abstract:**

In recent decades, biodiversity loss has greatly impacted planetary and human health. Children are at additional risk of adverse effects due to unique biological, developmental, and behavioral factors, as well as their longer exposure to an altered planet as a function of their young age. These effects are heightened for children living in vulnerable socioeconomic conditions. Here, we review the role of biodiversity loss on accelerating the consequences of climate change from the perspective of pediatric health. With the loss of biodiversity’s protective role against the consequences of climate change, the adverse effects of the changing planet are impacting pediatric health. For example, trees provide shelter against heat waves, unsealed soil and wetlands mitigate flooding, and rewilded green space hosts high microbial richness and consequently supports immune and mental health. The effects of the loss of biodiversity may impact the discovery and development of novel pharmaceuticals and thus the future of children’s medicine as a whole. We also highlight areas for further study and detail efforts that have been made to restore biodiversity, with the aim to improve the current and future health of local pediatric populations.

**Impact:**

- Loss of biodiversity is occurring at a rapid pace affecting the health of the planet and disproportionately pediatric health.
- This paper describes the role of biodiversity loss in accelerating the impact of climate change on children’s health, and highlights particularly vulnerable populations.
- This paper details steps that can be taken to maintain and restore biodiversity at the local and global levels to protect these populations and pediatric health in general.

## Introduction

Biodiversity refers to the variability among living organisms and the ecosystems in which they live.^1^ Healthy children rely on biodiverse and well-functioning ecosystems for a large number of reasons, including but not limited to producing oxygen, sequestering atmospheric carbon, building organic topsoil, preventing floods, and pollinating crops. Unfortunately, biodiversity on Earth is in a precipitous decline. There has been approximately a 69% decrease in biodiversity as estimated by monitored wildlife populations, around the globe since 1970.^2^ A recent report found that more than a third of species and ecosystems in the United States are at risk of disappearing, including 34% of plant species, 40% of animal species and 41% of ecosystems.^3^ Loss of biodiversity is accelerating the consequences of climate change and directly impacts pediatric health.

**Fig. 1:**
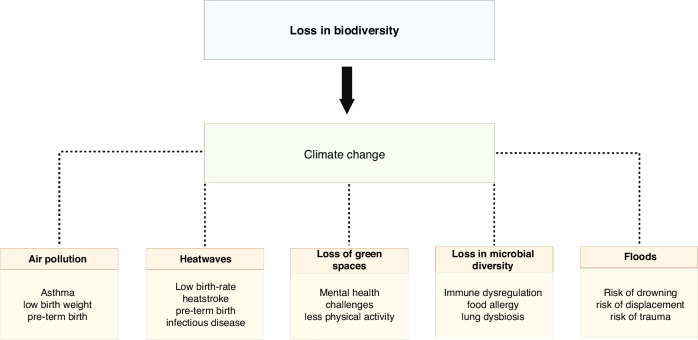
The various nodes of biodiversity that ultimately affect pediatric health outcomes throughout the globe.

The loss of biodiversity is advancing climate change with devastating impacts on human health, and a disproportionate effect on children. Climate change affects child health through its effects on microbial diversity, green space, average global temperature, and air pollution with a larger effect on especially vulnerable populations.^4^ Fig. 1 summarizes the accelerated effects of climate change due to the loss biodiversity that are adversely affecting the health of children. This paper will explore how these various nodes directly affect the future of children’s healthcare. This review paper summarizes the available published literature on the impact of a loss of biodiversity on pediatric health outcomes. Literature included represents both qualitative and qualitative studies, summarizing the varied and diverse ways in which biodiversity influences pediatric health.

## Loss of microbial diversity

There is strong evidence to suggest that biodiversity plays a direct role in the development of the immune system and the development of food allergies and respiratory infection. Biodiversity loss, including loss of microbial diversity, has been associated with a higher prevalence of allergic and inflammatory disorders. ^5^ The “biodiversity hypothesis” states that contact with natural environments enriches the human microbiome, promotes immune balance, and protects from allergy and inflammatory disorders.^5^ The biodiversity hypothesis describes two sources of biodiversity; exogenous (soil, water, plants, animals) and endogenous (skin, gut, airways), which collectively influence immune health.^5^

Recent intervention trials among healthy children in daycare centers have supported the biodiversity hypothesis in demonstrating that daily exposure to microbial biodiversity is associated with immune regulation, such as a positive change in interleukin-10 (IL-10; an anti-inflammatory cytokine involved in immunologic tolerance) when children regularly touch biodiverse soil and vegetation.^6–8^ However, the geographic region of these trials is narrow, and the effect of biodiversity intervention trials on actual disease incidence is yet to be explored. The gap is partially filled with correlative studies on the availability of biodiversity and disease incidence.

In one study performed on 14- to 18-year-old children living in Finland, it was found that atopic individuals had much lower environmental biodiversity around their homes, and this was associated with lower diversity in the bacteria on their skin. Specifically, IL-10 was decreased in this population of atopic individuals as compared to healthy individuals.^9^ Another study analyzed four study cohorts of children between the ages of 6 months and 20 years in Finland and Estonia, and determined that the amount of forest and agricultural land surrounding the children’s homes was inversely correlated with atopic sensitization.^10^ The study, however, did not take into account site characteristics, such as park age or various vegetation layers, within a land cover type, neither did it consider the children’s unique interactions with green space. Interestingly, one study in Australia found that when researchers disturbed the skin microbiome of school children through sanitizing wipes, exposure to green spaces, and more broadly, increased biodiversity, enabled a quicker, fuller microbial recovery than when exposure was limited to the indoor classroom. A more diverse skin microbiota was found in those children who spent time (45 min) in a forest compared to those of their indoor peers, and the impacts compounded over time.^11^ Taken together, these studies suggests the significant benefit of close contact with microbial biodiversity has on immune regulation. Provided that both immunological and microbial changes persist, exposure to microbial diversity plausibly leads to preventing the development of allergies. We encourage long-term biodiversity intervention trials that focus on disease incidence to remove the uncertainty of correlative studies connecting biodiversity, atopic sensitization, and allergies.

The gastrointestinal tract (GI) or gut, harbors roughly 100 trillion microbes from at least 160 different species making up an internal microbiome that facilitates the mediation of the immune response and development of immune tolerance. Gut microbiome dysbiosis, a byproduct of decreased biodiversity, can also potentiate the development of allergic, inflammatory, and autoimmune diseases.^12^ Early microbiota colonization critically influences adaptive and innate immunity; disruption of microbiota composition is linked with allergy and immune dysfunction, impacting children and often preceding adulthood.^13^ A longitudinal development study revealed that infants with a lower microbial richness and an imbalance in specific microbiota (i.e., Enterobacteriaceae to Bacteroidaceae (E/B) ratio and Ruminococcaceae abundance) at 3 months of age were correlated with a twofold increase in the likelihood of food sensitization.^14^ By 12 months of age, food sensitization was no longer associated with gut richness, however, sensitized infants were identified by a higher E/B ratio and low Ruminococcaceae abundance. This emphasizes the criticality of early life exposure to exogenous sources of microbiota and reveals the potential association between gut richness and food allergy development.^14^

Studies utilizing fecal transplants and probiotic supplementation have shown promise in the prevention and treatment of allergic disease through the restoration of intestinal permeability, immunological barrier function, and microbiota biodiversity. Limitations of so-far published studies include that parent-reported instances of physician diagnosis of food allergy are often skin-test negative. The uncertainty of food allergy diagnosis at a young age requires longitudinal analysis to follow the persistence of food sensitization and clinical food allergy. Even when results indicate a causal association, environmental factors can also influence dysbiosis of the microbiota and food sensitization.^15^ However, from the viewpoint of biodiversity loss and climate change, fecal transplants and probiotics are not curative. Therefore, urban environments should facilitate and encourage daily close contacts with biodiversity.^16^

Additionally, lung and airway dysbiosis is associated with respiratory viral infection and disease pathogenesis such as asthma, chronic obstructive pulmonary disease, and cancer and is known to exacerbate case severity. Lung dysbiosis may influence disease severity in patients infected with severe acute respiratory syndrome coronavirus 2 (SARS-CoV-2), emphasizing the importance of maintaining optimal microbial content and its symbiotic relationship with the environment.^17^ Urban air microbiome is enriched by increasing nearby green space, but if this is impossible, indoor gardening and green walls have been utilized as temporary solutions.^18–20^

## Decrease in green space

Green spaces and visual contact of green space have been associated with greater mental health. In a systematic review of 296 studies on nature contact and children, strong evidence was found for a positive relationship between nature contact and children’s cognitive, behavioral, and mental health.^21^ Most of these studies demonstrated the positive relationship between nature contact and children’s attention and mood, suggesting that nature contact could help care for children with attention-deficit/hyperactivity disorder (ADHD) and mood disorders such as anxiety and depression.^21^ In an experimental study, classrooms that looked out onto natural landscapes were shown to reduce stress and improve attention in high school students.^22^ Other studies have demonstrated reduced physiologic stress in adults when exposed to greater plant species diversity, as well as greater varieties in bird species.^23–27^

Several studies have suggested that areas of nature with high biodiversity may be associated with greater restorative capabilities when recovering from surgery or illness.^28^ Proximity to green space has been linked to improved pediatric respiratory health through reduction of wheezing and bronchitis.^28^ In adults, studies have found that patients with hospital windows looking out on a natural scene recovered faster from surgery than those facing a brick wall. In other studies, indoor plants reduced hospitalization times, pain, and frequency of use of pain medications.^26^ Current research findings needs to be supplemented with more non-self-report data, as many published studies consist of observational and cross-sectional data that focus primarily on adults, rather than children specifically.

Nature contact also encourages healthy behaviors in children, including increased physical activity leading to weight management and cardiorespiratory fitness. Fyfe-Johnson et al. found that 71 out of 88 observational studies demonstrated a positive association between nature contact and physical activity for children. A large study found that children living in neighborhoods without a park were more likely to be obese, overweight, physically inactive, and obtain inadequate sleep.^29^ In another study, the efficacy of gardening interventions was investigated in low-income elementary schools. Children in gardening programs had increased physical activity as compared to those in control schools.^21^ In another intervention trial among 3-5-year-old daycare children, transforming standard yards into biodiverse green spaces afforded well-being, play, and environmental relationships.^30^ Some studies in adults have demonstrated increased self-reported physical activity in environments that had more plant species.^31^

To summarize, increasing the amount of green space, vegetation diversity, and soil contacts not only encourages healthy behavior, but is restorative and helps improve the mental health of children. Although evidence of benefits is compelling, large intervention trials, consisting of thousands of children participating in greening and rewilding interventions would facilitate in-depth analysis of the benefits of green space on children.

## Average global temperature: heat waves and flooding

It is estimated that climate change, accentuated by biodiversity loss, has contributed to an average global temperature increase of 1.1 °C between 2011 and 2020 compared to 1850–1900s and increased frequency of extreme weather events such as heat waves.^32^ The stress of extreme heat exposure increases children’s risk of adverse events. There is evidence that children suffer negative impacts of extreme heat before they are born; extreme heat has been associated with increased rates of preterm birth, higher likelihood of low birthweight or stillborn births, especially in women in a lower socioeconomic class. Heat stress is also cited as increasing the incidence of sudden infant death syndrome.^33^ Infants are unable to effectively thermoregulate, and older children spend more time outdoors than adults and thus are more prone to the stress of heat waves.^34^ Heat waves are also cited as increasing the risk of children experiencing heat stroke, dehydration, and decreased learning.^35–37^ Biodiversity per se does not protect from heat waves, but climate-sensitive urban planning that integrates vegetation, and particularly trees and forests, into the built environment can mitigate the consequences of extreme heat waves.^38^

Other extreme weather events, such as severe flooding and storms are estimated to have increasing impacts on child health as the 21st century progresses; storms and flooding increase the risk of drowning, displacement, psychological trauma, and subsequent mental health sequelae. In the United States, flood risk is estimated to increase by 26.4% by 2050.^39^ As with extreme heat, the negative impacts will be felt first and most fiercely in low-income communities.^39^ Restoration of ecosystems along shorelines and rivers through the creation of estuaries and wetlands can attenuate impacts of severe flooding, storm waves, landslides, and to some extent sea level rise, thus buffering against health consequences for children.^40–42^

## Average global temperature: infectious diseases

A warming world has led to increases in the geographical spread of numerous vector-borne diseases, such as dengue fever and Zika virus, as mosquitoes and other vectors increase their habitat range.^43^ The WHO claimed that from 2021 to 2022 there was a twofold increase in dengue cases reported in the Americas and a similar trend for chikungunya.^44,45^ Compared to general population, children are more susceptible to many infectious diseases, due to their immature immune systems. While local efforts to decrease mean annual temperature are limited, biological agents against insect vectors have been developed, e.g., against *Aedes aegypti*, the vector of Zika and dengue viruses.

As climate change progresses, the duration for which environments are favorable for vectors of infectious disease is increasing. For example, the 2023 EPA Clinician Summary on Climate Change and Children’s Health and Well-Being in the United States, reports a 31% increase in new cases Lyme Borreliosis among children in the Eastern U.S. at an average temperature increase of 2°C. At higher temperature increases around 4°C, the concern is for a 272% increase of case incidence above baseline.^4^ Lyme Borreliosis is spread via an infected tick. Unlike tropical diseases, ticks do not need a specific vector but use numerous terrestrial vertebrates as hosts. Therefore, they might even become more common as a result of ecosystem restoration, despite measures to decrease the warming world. The best practice is to prevent tick bites through individual management and monitoring, such as wearing proper cloths and checking for tick bites.^46^ However, the incidence of tick-borne diseases is magnitudes lower than the incidence of non-communicable immune-mediated diseases.^47,48^ So far, an integrated approach that considers eco-bio-social determinants of the virus-vector-man chain has been the most successful in limiting the negative effects of vector-borne diseases on pediatric health.^49^ This underlines the need for further investments in holistic research initiatives and action plans.

## Air pollution

Air pollution is closely linked to biodiversity loss because different species withstand pollution differently, and because many pollutants that spread in air, eventually will accumulate in soil. Together with consequences of climate change, such as stressful heat waves, air pollution risks are particularly felt among vulnerable populations that are unable to live outside polluted areas. According to the WHO, at least 1.5 million children under the age of 5 years die annually due to air and water pollution and exposure to toxic substances.^50^ Air pollution has been linked to the development of several non-communicable diseases in childhood, such as asthma. Children’s frequent hand-to-mouth behavior contributes to their increased risk of exposure to pollutants. As children drink, eat, and breathe more than adults relative to their body weight, they ingest a higher proportion of pollutants and toxins found in food, air, and water.^51^ Also, children’s increased longitudinal exposure to environmental pollutants, due to their younger age and longer lifetime exposure, results in greater health complications as compared to adults. Further, their immune and hepatorenal systems are still developing, making them more susceptible to pollutants while being less able to excrete toxins.^52^ Toxins such as lead, mercury, and others are known to have negative, long-term impacts on developing organ systems. For example, in utero exposure to wildfire smoke has been associated with decreased birth weight and preterm birth.^51^ Maternal exposure to polycyclic aromatic hydrocarbons (PAH), which are attributed to the burning of biomass and fossil fuels, vehicular emissions, and wildfires, have been associated with in utero epigenetic effects on the fetus that later predisposes the child to asthma. Similarly, exposure to PAHs negatively impacts IFN-γ, a gene strongly correlated with a protective mechanism associated with allergy and asthma.^53^

The most effective approach to prevent the negative consequences of air pollution on pediatric health is to reduce the emission of pollutants. Another possibility is to remediate already polluted water, air and soil. Soil remediation can be done in situ, which saves at least some of the local biodiversity,^54,55^ or polluted soil can be excavated and remediated or capsulated elsewhere. In situ remediation is time-consuming, while excavation requires considerable economic resources. Forests can filtrate air pollutants efficiently, but the filtration occurs mostly in sparsely populated areas.^56^ Thus far research has found it to be inefficient in the context of pediatric health. Similarly, while trees have traditionally been assumed to increase air quality, a recent reassessment of the effects of vegetation on urban air quality concluded that urban vegetation is inefficient in pollution removal, that trees can even weaken the dispersal of pollutants from street level, and that the main focus in enhancing air quality must be pollution prevention, not filtration.^57^ From the pediatric health perspective, since children’s daily contacts with biodiverse vegetation enrich commensal microbiota and enhance immune regulation,^6–8^ rewilding living environment plausibly generates other benefits also in polluted urban environment. For example, locally developed urban forests have been associated with increased gut health^58^ therefore despite the lack of evidence supporting air pollution filtration, increasing amount of vegetation and trees in urban environments still have beneficial pediatric health outcomes.

## Loss of biodiversity on pediatric medicine

Biodiversity is critical in the discovery and development of novel pharmaceuticals. Many medicines in use today rely on plants thriving in well-functioning ecosystems.^59^ The WHO estimates that 11% of essential medicines are derived from plants, whereas other studies have found that the proportion of antibacterial, antiviral, and antiparasitic medications in the United States derived from plants is closer to 75%.^60,61^ Further, much of the world continues to depend on traditional medicines derived from plants.^62^ As these ecosystems are destroyed, so are the plants enabling the manufacture of medications. Estimates suggest that due to biodiversity loss, the discovery of at least one important drug is missed every two years.^63^ Scarcities in well-established medicines as well as a decline in the discovery of therapeutic options will plausible have a negative impact on children, who may have less access to critical medicines in the future than we do today.

## Ecosystem restoration as a mitigation strategy

Ecosystem restoration and particularly rewilding i.e. establishing biologically diverse ecosystems to otherwise non-biodiverse neighborhoods, is a highly promising strategy to mitigate many of the effects of biodiversity loss and provide ecosystem services for children. For example, vegetation can be utilized to provide shelter from extreme temperatures while mitigating flooding. Hence, restoring biodiversity will be critical in the 21st century. The UN has declared 2021–2030 to be the Decade on Ecosystem Restoration and has called for countries to “halt and reverse the degradation of ecosystems worldwide and raise awareness of the importance of successful ecosystem restoration”.^64^ According to the UN, reviving ecosystems around the globe could potentially contribute to around one-third of the climate change mitigation needed by 2030, reduce the risk of future pandemics, and increase food security for at least 1.3 billion people.^65^

Ecosystem restoration and rewilding cover many different actions. On agricultural land, sustainable agricultural practices that will aid in restoring healthy ecosystems include agroforestry, organic farming, and climate-smart and conservation farming practices. These practices will help in decreasing soil erosion, increase carbon storage in soils, and replenish crop microbial diversity.^65^ Restoration along coastlines can decrease the impacts of agricultural run-off on water quality and marine ecosystems. As an example, according to the Mangrove Restoration Potential Map developed by the University of Cambridge, restoring mangroves globally could add over 60 trillion young fish to coastal waters annually,^66^ which aids in nourishing vulnerable pediatric populations. In urban areas, where most children on Earth reside, ecosystem restoration and rewilding should be done synergistically to maximize health benefits and overall biodiversity restoration.

The loss of biodiversity is increasingly becoming a child rights crisis, threatening the health and safety of children around the globe. Certain groups of children, including disabled, refugees and other immigrant communities, those experiencing housing instability, and those separated from their family members are at increased risk of the adverse health effects of climate change and biodiversity loss. Ecosystem restoration projects that seek to engage children directly are therefore an area of future research. As further research and action are being done, children should be directly involved in the development and creation of policies and interventions. For example, an advisory service in Berlin, Germany is working collaboratively with children, to rewild school and kindergarten yards. The mission of the organization is to let pupils and children design, plan and implement their ideas to learn how to develop and preserve natural resources in sustainable manners.^67^ The work by advisory service in Berlin serves as a model in children’s engagement in decision-making; the engagement is central to the United Nations Sustainable Development Goals 13 – Climate Action, 15 – Life of Land, and 17 – Partnerships for the Globe. Given the worsening mental health crisis in youth in the United States over the past ten years, developing ways for children to become active participants in solutions to larger environmental problems will likely have health co-benefits when considering protective factors against mental health issues, including self-efficacy, a sense of agency and community, and time spent in nature.

## Limitations and future directions

Although this review has emphasized the key roles biodiversity plays in children’s health, several research limitations must be addressed. Currently, most of the existing research heavily relies on cross-sectional and observational data which may not yield conclusive evidence of causality. For example, most research often focused only on specific geographic regions; revealing gaps in our understanding of how global loss of biodiversity affects child health, especially those in underserved populations. Current research gaps include the benefits versus risks of diet containing soil microbiota,^68^ the causal effect of rich, non-harmful environmental microbiota on disease incidence,^20^ and the mutual benefits of ecosystem restoration and rewilding of grey urban space on humans, ecosystems, and carbon sequestration.

Furthermore, there is a large data gap regarding longitudinal studies tracking the long-term health effect of biodiversity interventions, which are crucial in assessing the sustained impact on disease incidence and overall well-being. Most studies on microbial diversity and green space exposure don’t consider the complex interactions between socioeconomic factors, health inequities, and urbanization, which also influence child health outcomes. This need for holistic, diverse, large-scale longitudinal studies is vital; understanding the interconnections between people, specifically children, and animals, plants, microbes, and our shared environment, is important for the future direction of the well-being of children and our planet. Taking a collaborative, multi-sectoral, and global perspective to understand the multifaceted relationship between these complex and important topics is imperative to understanding the effects of biodiversity on health.

## Conclusion

We are in a period of human history during which we are seeing unprecedented loss of biodiversity which is taking a toll on planetary and human health. This review paper evaluated how a loss of biodiversity in the warming world is affecting microbial diversity, green space, heat waves, flooding, vector-borne diseases, and air pollution which ultimately affects the health of children. Specifically, we found that an increase in biodiversity is protective against immune and mental health. In parallel, even though biodiversity does not prevent climate change, adding biodiverse urban ecosystems has the potential to mitigate local problems caused by climate change. In short, biodiversity protects against the adverse effects of climate change and its absence is impacting child health, especially vulnerable pediatric populations.

Today, systematic holistic studies on how ecosystem restoration and rewilding are enhancing everyday life of children are scarce. It will be particularly important to prioritize research on children, as their growth and development make them intrinsically different from adults. Based on the research provided, future interventions to address loss of biodiversity benefits from a multidisciplinary focus, considering not only how to reduce the impact of climate change on children’s health, but how to leverage biodiversity to improve the health outcome of children globally. We need individual, local and multi-country interventions and responses to address and mitigate this serious threat to human, specifically, child life. This necessitates the engagement of the local community, government, school systems, and academic institutions, not only those involved in human health, but also those focusing on social aspects, ecosystems, and urban planning. However, despite the lack of large scale systemic studies, there is sufficient evidence to support the need for increased biodiversity among urban environments to support the health and wellbeing of children globally.

## References

[1] United Nations. Convention on Biological Diversity. (1992). 10.1016/s0378-8741(96)90036-7 9213623

[2] World wildlife fund. Living Planet Report. https://livingplanet.panda.org/en-US/ (2022).

[3] NatureServe. Biodiversity in Focus: United States. (2023).

[4] Concept note: General comment on children’s rights and the environment with a special focus on climate change. *OHCHR* https://www.ohchr.org/en/treaty-bodies/crc/concept-note-general-comment-childrens-rights-and-environment-special-focus-climate-change.

[5] Haahtela, T. A biodiversity hypothesis. *Allergy* **74**, 1445–1456 (2019). 30835837 10.1111/all.13763

[6] Roslund, M. I. et al. Biodiversity intervention enhances immune regulation and health-associated commensal microbiota among daycare children. *Sci. Adv.* **6**, eaba2578 (2020). 33055153 10.1126/sciadv.aba2578 PMC7556828

[7] Roslund, M. I. et al. A Placebo-controlled double-blinded test of the biodiversity hypothesis of immune-mediated diseases: Environmental microbial diversity elicits changes in cytokines and increase in T regulatory cells in young children. *Ecotoxicol. Environ. Saf.* **242**, 113900 (2022). 35930838 10.1016/j.ecoenv.2022.113900

[8] Roslund, M. I. et al. Long-term biodiversity intervention shapes health-associated commensal microbiota among urban day-care children. *Environ. Int.* **157**, 106811 (2021). 34403882 10.1016/j.envint.2021.106811

[9] Hanski, I. et al. Environmental biodiversity, human microbiota, and allergy are interrelated. *Proc. Natl. Acad. Sci.* **109**, 8334–8339 (2012). 22566627 10.1073/pnas.1205624109 PMC3361383

[10] Ruokolainen, L. et al. Green areas around homes reduce atopic sensitization in children. *Allergy* **70**, 195–202 (2015). 10.1111/all.12545 PMC4303942 25388016

[11] Mills, J. G., Selway, C. A., Thomas, T., Weyrich, L. S. & Lowe, A. J. Schoolyard biodiversity determines short-term recovery of disturbed skin microbiota in children. *Micro. Ecol.* **86**, 658–669 (2023). 10.1007/s00248-022-02052-2 PMC9188306 35689685

[12] Ray, C. & Ming, X. Climate change and human health: a review of allergies, autoimmunity and the microbiome. *Int. J. Environ. Res. Public Health* **17**, 4814 (2020). 32635435 10.3390/ijerph17134814 PMC7369820

[13] Akagawa, S. & Kaneko, K. Gut microbiota and allergic diseases in children. *Allergol. Int.* **71**, 301–309 (2022). 35314107 10.1016/j.alit.2022.02.004

[14] Azad, M. B. et al. Infant gut microbiota and food sensitization: associations in the first year of life. *Clin. Exp. Allergy* **45**, 632–643 (2015). 25599982 10.1111/cea.12487

[15] Lee, K. H., Song, Y., Wu, W., Yu, K. & Zhang, G. The gut microbiota, environmental factors, and links to the development of food allergy. *Clin. Mol. Allergy* **18**, 5 (2020). 32265597 10.1186/s12948-020-00120-x PMC7119288

[16] Sinkkonen, A. Evolution, Biodiversity and a Reassessment of the Hygiene Hypothesis. in *Evolution, Biodiversity and a Reassessment of the Hygiene Hypothesis*. (Springer International Publishing, Cham, 2022). 10.1007/978-3-030-91051-8.

[17] Khatiwada, S. & Subedi, A. Lung microbiome and coronavirus disease 2019 (COVID-19): Possible link and implications. *Hum. Micro. J.* **17**, 100073 (2020). 10.1016/j.humic.2020.100073 PMC7405772 32835135

[18] Robinson, J. M. & Breed, M. F. The aerobiome-health axis: a paradigm shift in bioaerosol thinking. *Trends Microbiol* **31**, 661–664 (2023). 37211511 10.1016/j.tim.2023.04.007

[19] Soininen, L. et al. Indoor green wall affects health-associated commensal skin microbiota and enhances immune regulation: a randomized trial among urban office workers. *Sci. Rep.* **12**, 6518 (2022). 35444249 10.1038/s41598-022-10432-4 PMC9021224

[20] Saarenpää, M. et al. Urban indoor gardening enhances immune regulation and diversifies skin microbiota — A placebo-controlled double-blinded intervention study. *Environ. Int.* **187**, 108705 (2024). 38688234 10.1016/j.envint.2024.108705

[21] Fyfe-Johnson, A. L. et al. Nature and children’s health: a systematic review. *Pediatrics* **148**, e2020049155 (2021). 34588297 10.1542/peds.2020-049155

[22] Li, D. & Sullivan, W. Impact of views to school landscapes on recovery from stress and mental fatigue. *Landsc. Urban Plan.* **148**, 149–158 (2016).

[23] Wolf, L. J., Ermgassen, S,-Z., Balmford, A., White, M. & Weinstein, N. Is Variety the Spice of Life? An experimental investigation into the effects of species richness on self-reported mental well-being. *PLoS ONE* **12**, e0170225 (2017). 28107417 10.1371/journal.pone.0170225 PMC5249088

[24] Johansson, M., Gyllin, M., Witzell, J. & Küller, M. Does biological quality matter? Direct and reflected appraisal of biodiversity in temperate deciduous broad-leaf forest. *Urban For. Urban Green.* **13**, 13–28 (2014).

[25] Cox, D. et al. Doses of neighborhood nature: the benefits for mental health of living with nature. *BioScience* **67**, 147–155 (2017).

[26] Lindemann-Matthies, P. & Matthies, D. The influence of plant species richness on stress recovery of humans. *Web Ecol.* **18**, 121–128 (2018).

[27] Schebella, M. F., Weber, D., Schultz, L. & Weinstein, P. The wellbeing benefits associated with perceived and measured biodiversity in Australian urban green spaces. *Sustainability* **11**, 802 (2019).

[28] Lucey, M. Urban biodiversity affects children’s respiratory health. *Lancet Respir. Med* **5**, 613 (2017). 28748807 10.1016/S2213-2600(17)30272-2

[29] Reuben, A., Rutherford, G., James, J. & Razani, N. Association of neighborhood parks with child health in the United States. *Prevent. Med.* **141**, 106265 (2020). 10.1016/j.ypmed.2020.106265 PMC8034548 33035547

[30] Puhakka, R. et al. Greening of daycare yards with biodiverse materials affords well-being, play and environmental relationships. *Int. J. Environ. Res Public Health* **16**, 2948 (2019). 31426345 10.3390/ijerph16162948 PMC6719197

[31] de Jong, K., Albin, M., Skärbäck, E., Grahn, P. & Björk, J. Perceived green qualities were associated with neighborhood satisfaction, physical activity, and general health: Results from a cross-sectional study in suburban and rural Scania, southern Sweden. *Health Place* **18**, 1374–1380 (2012). 22889998 10.1016/j.healthplace.2012.07.001

[32] *Climate Change 2022: Impacts, Adaptation and Vulnerability. Contribution of Working Group II to the Sixth Assessment Report of the Intergovernmental Panel on Climate Change*. (2022).

[33] Chersich, M. F. et al. Associations between high temperatures in pregnancy and risk of preterm birth, low birth weight, and stillbirths: systematic review and meta-analysis. *BMJ* **371**, m3811 (2020). 33148618 10.1136/bmj.m3811 PMC7610201

[34] Smith, C. J. Pediatric thermoregulation: considerations in the face of global climate change. *Nutrients* **11**, 2010 (2019). 31454933 10.3390/nu11092010 PMC6770410

[35] Hsu, A., Sheriff, G., Chakraborty, T. & Manya, D. Disproportionate exposure to urban heat island intensity across major US cities. *Nat. Commun.* **12**, 2721 (2021). 34035248 10.1038/s41467-021-22799-5 PMC8149665

[36] Bekkar, B., Pacheco, S., Basu, R. & DeNicola, N. Association of air pollution and heat exposure with preterm birth, low birth weight, and stillbirth in the US: a systematic review. *JAMA Netw. Open* **3**, e208243 (2020). 32556259 10.1001/jamanetworkopen.2020.8243 PMC7303808

[37] Auger, N., Fraser, W., Smargiassi, A. & Kosatsky, T. Ambient heat and sudden infant death: a case-crossover study spanning 30 years in Montreal, Canada. *Environ. Health Perspect.* **123**, 712–716 (2015). 25748025 10.1289/ehp.1307960 PMC4492261

[38] Uittenbroek, C. J., Janssen-Jansen, L. B. & Runhaar, H. A. C. Mainstreaming climate adaptation into urban planning: overcoming barriers, seizing opportunities and evaluating the results in two Dutch case studies. *Reg. Environ. Change* **13**, 399–411 (2013).

[39] Wing, O. E. J. et al. Inequitable patterns of US flood risk in the Anthropocene. *Nat. Clim. Chang.* **12**, 156–162 (2022).

[40] Temmerman, S. et al. Ecosystem-based coastal defence in the face of global change. *Nature* **504**, 79–83 (2013). 24305151 10.1038/nature12859

[41] Carter, J., Handley, J., Butlin, T. & Gill, S. Adapting cities to climate change – exploring the flood risk management role of green infrastructure landscapes. *J. Environ. Plan. Manag.* **61**, 1–18 (2017).

[42] Miura, S. et al. Protective functions and ecosystem services of global forests in the past quarter-century. *For. Ecol. Manag.* **352**, 35–46 (2015).

[43] Williams, P. C. et al. Impact of climate change and biodiversity collapse on the global emergence and spread of infectious diseases. *J. Paediatr. Child Health* **57**, 1811–1818 (2021). 34792238 10.1111/jpc.15681

[44] Geographical expansion of cases of dengue and chikungunya beyond the historical areas of transmission in the Region of the Americas. https://www.who.int/emergencies/disease-outbreak-news/item/2023-DON448.

[45] Reaser, J. K., Witt, A., Tabor, G. M., Hudson, P. J. & Plowright, R. K. Ecological countermeasures for preventing zoonotic disease outbreaks: when ecological restoration is a human health imperative. *Restor. Ecol.* **29**, e13357 (2021). 33785998 10.1111/rec.13357 PMC7995086

[46] Köhler, C. F., Holding, M. L., Sprong, H., Jansen, P. A. & Esser, H. J. Biodiversity in the Lyme-light: ecological restoration and tick-borne diseases in Europe. *Trends Parasitol.* **39**, 373–385 (2023). 36890021 10.1016/j.pt.2023.02.005

[47] Wu, D. et al. Global, regional, and national incidence of six major immune-mediated inflammatory diseases: findings from the global burden of disease study 2019. *eClinicalMedicine* **64**, 102193 (2023). 37731935 10.1016/j.eclinm.2023.102193 PMC10507198

[48] Burn, L. et al. Incidence of Lyme Borreliosis in Europe: A Systematic Review (2005–2020) | Vector-Borne and Zoonotic Diseases. 10.1089/vbz.2022.0070. 10.1089/vbz.2022.0070 PMC10122234 37071407

[49] Lima, E. P., Goulart, M. O. F. & Rolim Neto, M. L. Meta-analysis of studies on chemical, physical and biological agents in the control of Aedes aegypti. *BMC Public Health* **15**, 858 (2015). 26341708 10.1186/s12889-015-2199-y PMC4559884

[50] Preventing disease through healthy environments: a global assessment of the burden of disease from environmental risks. https://www.who.int/publications/i/item/9789241565196.

[51] Carroquino, M. J., Posada, M. & Landrigan, P. J. Environmental Toxicology: Children at Risk. *Environ. Toxicol.* 239–291 (2012) 10.1007/978-1-4614-5764-0_11.

[52] Perera, F. & Nadeau, K. Climate Change, Fossil-Fuel Pollution, and Children’s Health | New England Journal of Medicine. https://www.nejm.org/doi/full/10.1056/NEJMra2117706. 10.1056/NEJMra2117706 35704482

[53] Syed, A., Hew, K., Kohli, A., Knowlton, G. & Nadeau, K. Air pollution and epigenetics. *J. Environ. Prot.* **47**, 151838 (2013).

[54] Cai, Z. et al. In situ electrokinetic (EK) remediation of the total and plant available cadmium (Cd) in paddy agricultural soil using low voltage gradients at pilot and full scales. *Sci. Total Environ.* **785**, 147277 (2021). 33957583 10.1016/j.scitotenv.2021.147277

[55] Romantschuk, M. et al. Bioremediation of contaminated soil and groundwater by in situ biostimulation. *Front. Microbiol.* **14**, 1258148 (2023). 10.3389/fmicb.2023.1258148 PMC10658714 38029190

[56] Nowak, D. J., Hirabayashi, S., Bodine, A. & Greenfield, E. Tree and forest effects on air quality and human health in the United States. *Environ. Pollut.* **193**, 119–129 (2014). 25016465 10.1016/j.envpol.2014.05.028

[57] Venter, Z. S., Hassani, A., Stange, E., Schneider, P. & Castell, N. Reassessing the role of urban green space in air pollution control | PNAS. *Proc. Natl. Acad. Sci.* **121**, e2306200121 (2024). 10.1073/pnas.2306200121 PMC10861851 38285938

[58] Vari, H. K. et al. Associations between land cover categories, gaseous PAH levels in ambient air and endocrine signaling predicted from gut bacterial metagenome of the elderly. *Chemosphere* **265**, 128965 (2021). 33248729 10.1016/j.chemosphere.2020.128965

[59] Howes, M.-J. R. The evolution of anticancer drug discovery from plants. *Lancet Oncol.* **19**, 293–294 (2018). 29508748 10.1016/S1470-2045(18)30136-0

[60] WHO Model Lists of Essential Medicines. https://www.who.int/groups/expert-committee-on-selection-and-use-of-essential-medicines/essential-medicines-lists.

[61] Newman, D. & Cragg, G. Natural products as sources of new drugs over the 30 years from 1981 to 2010. *J. Nat. Products* **75**, 311–335 (2012). 10.1021/np200906s PMC3721181 22316239

[62] Ekor, M. The growing use of herbal medicines: issues relating to adverse reactions and challenges in monitoring safety. *Front. Pharm.* **4**, 177 (2014). 10.3389/fphar.2013.00177 PMC3887317 24454289

[63] Neergheen-Bhujun, V. et al. Biodiversity, drug discovery, and the future of global health: Introducing the biodiversity to biomedicine consortium, a call to action. *J. Glob. Health* **7**, 020304 (2017). 10.7189/jogh.07.020304 PMC5735771 29302312

[64] United Nations General Assembly. Resolution Adopted by the General Assembly on 1 March 2019. (2019).

[65] United Nations Environment Programme - 2021 - Ecosystem Restoration for People, Nature and Clima.pdf.

[66] Worthington, T. & Spalding, M. Mangrove Restoration Potential: A global map highlighting a critical opportunity. (2018) 10.17863/CAM.39153.

[67] Sicard, P. et al. Should we see urban trees as effective solutions to reduce increasing ozone levels in cities? *Environ. Pollut.* **243**, 163–176 (2018). 30172122 10.1016/j.envpol.2018.08.049

[68] Roslund, M. I., Laitinen, O. H. & Sinkkonen, A. Scoping review on soil microbiome and gut health—Are soil microorganisms missing from the planetary health plate? *People Nat.* **6**, 1078–1095 (2024).

